# A High Geriatric Depression Scale Score on Admission to Hospital Predicts a Worse Clinical Frailty Scale Score After Discharge

**DOI:** 10.1111/ggi.70598

**Published:** 2026-06-30

**Authors:** Yosuke Yamada, Hirotaka Nakashima, Masaaki Nagae, Kazuhisa Watanabe, Chisato Fujisawa, Hitoshi Komiya, Tomihiko Tajima, Shosuke Satake, Yasushi Takeya, Yumi Umeda‐Kameyama, Hiroyuki Umegaki

**Affiliations:** ^1^ Department of Community Healthcare and Geriatrics, Graduate School of Medicine Nagoya University Nagoya Japan; ^2^ Department of Geriatric Medicine National Center for Geriatrics and Gerontology Obu Japan; ^3^ Department of Evidence‐Based Clinical Nursing, Division of Health Sciences, Graduate School of Medicine Osaka University Osaka Japan; ^4^ Dementia Center The University of Tokyo Hospital Tokyo Japan

**Keywords:** acute hospitalization, clinical frailty scale, depression, frailty, post‐discharge

## Abstract

**Aim:**

This study aimed to determine whether a higher Geriatric Depression Scale‐15 (GDS‐15) score on admission is associated with worsening of the Clinical Frailty Scale (CFS) score from discharge to 3 months after discharge in older patients hospitalized for acute care.

**Methods:**

This multicenter prospective study was performed at three university hospitals and one national medical center in Japan between October 2019 and July 2023. A total of 601 patients were analyzed. Post‐discharge CFS worsening was defined as a ≥ 1‐point increase in CFS score from discharge to 3 months after discharge. Logistic regression, subgroup, sensitivity, and formal interaction analyses were performed.

**Results:**

From discharge to 3 months after discharge, the CFS score worsened in 87 patients (14.5%). The worsened group had significantly higher GDS‐15 scores on admission (*p* = 0.047). After adjustment for age, sex, baseline CFS score, CCI value, and MMSE score, a higher GDS‐15 score was associated with greater odds of CFS worsening (OR 1.109, *p* = 0.002). In subgroup analysis, this association was observed in patients with baseline CFS scores ≥ 4 (OR 1.106, *p* = 0.007), but not in those with baseline CFS scores ≤ 3. The formal interaction test was not significant (*p* = 0.969). The association remained significant in sensitivity analyses.

**Conclusions:**

A higher GDS‐15 score on admission independently predicted post‐discharge CFS worsening in older acute‐care inpatients. GDS‐15 screening on admission may help identify patients requiring closer post‐discharge monitoring for frailty deterioration.

## Graphical abstract

Higher Geriatric Depression Scale‐15 scores on admission were associated with worsening of the Clinical Frailty Scale score from discharge to 3 months after discharge among older inpatients in geriatric wards.

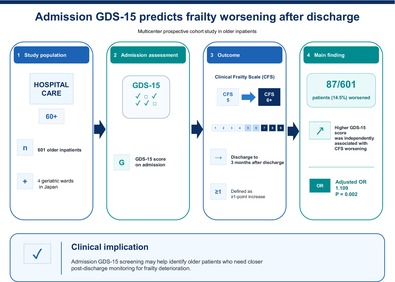

## 1 Introduction

In an aging society, the number of older people who require hospitalization continues to increase. It has been reported that 40% of older hospitalized patients are frail [1]. Frailty is defined as a state of increased vulnerability and decreased resistance to various stresses as a result of age‐related loss of physiological reserve [1]. These patients are at particularly high risk for adverse outcomes during hospitalization and after discharge [2]. While it is important to prevent functional decline and deterioration of frailty during hospitalization in order to remain independent at home for longer, it is equally important to prevent deterioration after discharge. In many cases, physical function improves after discharge from acute hospitalization, even in older patients [3]. A report on patients over 65 years of age admitted to a university hospital showed that the proportion of frail patients decreased from 50.6% at discharge to 47.9% 3 months later [4]. However, after discharge from an acute hospitalization, there are a small number of patients whose physical function and frailty are worse than at the time of admission. In a report on 4504 Canadian seniors hospitalized for acute respiratory illness, approximately 57% were less frail and approximately 9% were more frail at 1 month after discharge than at the time of admission [5]. However, there has been little research on which patients are more likely to have worsening frailty after discharge.

Depression is one of the most common psychiatric symptoms in older people, with one study finding that 17.2% of those aged 75 years or older had a depressive disorder [6]. Depression is associated with higher rates of comorbid physical illness in older adults than in their younger counterparts and is associated with cognitive impairment and physical decline [7, 8]. Furthermore, depression has been reported to be more common in older adults and is often a more serious problem in both outpatient and inpatient hospital settings than in the community [9]. Depression in hospitalized older patients is associated with increased mortality [10], a longer hospital stay [11], and an increased likelihood of readmission [12]. Furthermore, symptoms of depression (e.g., anorexia, fatigue, and insomnia) often overlap with symptoms of physical illness, making inpatient care more complex and challenging [13].

It is well known that both frailty and depression are associated with a variety of adverse outcomes in older people [14]. Previous research has shown that depression and frailty share common risk factors and interact [15], and frailty has been found in approximately 40% of older people who become depressed in later life [16]. This high prevalence may be explained by common syndromes and pathophysiological pathways, including cognitive impairment, impaired ability to perform activities of daily living, decreased activity levels, social isolation, and chronic inflammation [15]. Therefore, depression and frailty are closely related. There have been reports suggesting that depression itself worsens frailty in older people [17] and that the presence of depression increases the incidence of frailty [17, 18]. However, the reports published to date have tended to focus on community‐dwelling older people and psychiatric or geriatric outpatients, with few focusing on acutely hospitalized or discharged patients. Prevention of worsening frailty in older patients discharged from acute hospitalization is one of the most important goals of inpatient care in geriatrics. Therefore, this study aimed to determine whether a higher Geriatric Depression Scale‐15 (GDS‐15) score on admission was associated with worsening of the Clinical Frailty Scale (CFS) score from discharge to 3 months after discharge in older patients hospitalized for acute care.

## 2 Material and Methods

### 2.1 Study Design

The data analyzed in this prospective cohort study were from the Japan Hospital Acquired Complications project, which is an observational multicenter study being performed in geriatric wards in Japanese hospitals. The present study included patients admitted to and discharged from an acute geriatric ward at any of three university hospitals or one national medical center in Japan between October 2019 and July 2023.

The inclusion criterion for this study was age 60 years or older. Exclusion criteria were a hospital stay of 2 days or less, lack of informed consent, an estimated life expectancy of less than 1 month in the opinion of the attending physician, readmission within 3 months of the last hospitalization, in‐hospital transfer, missing CFS data at baseline, discharge, or 3 months after discharge, missing GDS‐15 data on admission, or inability to complete the GDS‐15.

The study protocol was approved by the ethics committee at each participating facility. Informed consent was obtained from all participants.

### 2.2 Assessment of Depression

Depressive symptoms were assessed using the 15‐item Geriatric Depression Scale (GDS‐15) [19], which was administered by a physician or research assistant within 7 days of admission.

### 2.3 Assessment of Frailty

Frailty was assessed using the CFS, which is a 9‐point instrument developed by Rockwood et al. to screen for frailty in clinical practice and is associated with mortality, comorbidity, cognitive function, falls, and mobility function [20]. The CFS is now widely used internationally because of its simplicity and validity [21]. The CFS score was recorded on admission to hospital, at discharge, and 3 months after discharge by the physician in charge or a trained research assistant. The baseline CFS score was calculated based on information on frailty status 2 weeks before admission collected from patients or their caregivers at the time of admission to hospital. The 3‐month CFS assessment was conducted by telephone interview with patients or their caregivers. The same CFS scoring criteria were used at all participating centers.

### 2.4 Other Variables

Data on age, sex, education level, and the Charlson Comorbidity Index (CCI) value were collected from the clinical charts. Ability to perform basic activities of daily living (BADL) was assessed using the Barthel index [22] based on information obtained at the time of admission from patients or caregivers concerning the ability to perform these activities 2 weeks before admission. Cognitive function was assessed using the Mini‐Mental State Examination (MMSE) [23], which was administered by a physician or research assistant within 7 days of admission, as was nutritional status using the Mini Nutritional Assessment Short Form [24].

Data on emergency admission, length of hospital stay, living arrangement, congestive heart failure, cerebrovascular disease, and C‐reactive protein (CRP) level were collected from clinical charts or interviews with patients or caregivers, as appropriate. Living arrangement was categorized as living alone or not living alone.

### 2.5 Statistical Analysis

Post‐discharge worsening of the CFS score was defined as an increase of at least 1 point in the CFS score from discharge to 3 months after discharge. The participants were divided into two groups according to whether the CFS score worsened from discharge to 3 months after discharge. Differences between groups were compared using the chi‐squared test, *t*‐test, or Mann–Whitney U test, as appropriate. Multiple logistic regression analysis was performed with post‐discharge worsening of the CFS score as the objective variable and GDS‐15 score on admission as the predictor variable. Model 1 was adjusted for age and sex.

Model 2 was additionally adjusted for baseline CFS score. Model 3 was additionally adjusted for CCI value and MMSE score. The Barthel index and MNA‐SF score were not included in the main model because of their potential overlap with the frailty construct and were instead evaluated in sensitivity analyses. A subanalysis was performed using the group with a baseline CFS score of ≥ 4 as the frail group and the group with a baseline CFS score of ≤ 3 as the non‐frail group. To examine whether baseline frailty status modified the association between GDS‐15 score on admission and post‐discharge CFS worsening, an interaction analysis was performed by adding baseline frailty status and the interaction term between GDS‐15 score and baseline frailty status to the logistic regression model. Baseline frailty status was defined as a baseline CFS score of ≥ 4. The interaction model was adjusted for age, sex, CCI value, and MMSE score.

A sensitivity analysis was also performed in the frail group after excluding patients with an MMSE score of < 15 to examine the possibility that the GDS‐15 score might not be accurate in patients with advanced severe cognitive impairment. In the overall cohort, sensitivity analyses were performed by adding the following variables to Model 3: length of hospital stay; congestive heart failure and cerebrovascular disease; congestive heart failure, cerebrovascular disease, and length of hospital stay; living alone; Barthel index and MNA‐SF score; and emergency admission, length of hospital stay, and log‐transformed CRP. CRP was log‐transformed as ln(CRP + 1), and CRP values below zero were treated as missing. Variables with missing data were analyzed using available cases, and no imputation was performed.

Statistical analyses were performed using SPSS (version 29; IBM Corp., Armonk, NY, USA) and R (version 4.5.2; R Foundation for Statistical Computing, Vienna, Austria). A *p*‐value of < 0.05 was considered to indicate statistical significance.

## 3 Results

During the study period, 1848 patients were admitted to and discharged from any of the participating centers. After 1247 exclusions, 601 patients were included in the analysis. The study flowchart is shown in Figure S1. From discharge to 3 months after discharge, the CFS score worsened in 87 patients (14.5%) and was maintained or improved in 514 patients (85.5%). The participants' background characteristics are compared according to whether their CFS score worsened from discharge to 3 months after discharge in Table 1. The mean age was 83.6 ± 6.7 years, and 253 participants (42.1%) were male. The mean baseline CFS score was 5.0 ± 1.7 and the mean GDS‐15 score on admission was 4.9 ± 3.7. The group with a worsened CFS score had a significantly higher education level (*p* = 0.022), higher Barthel index (*p* = 0.008), and a higher GDS‐15 score on admission (*p* = 0.047). This group also had significantly lower baseline CFS scores (*p* = 0.003), lower CFS scores at discharge (*p* < 0.001), and significantly higher CFS scores at 3 months after discharge (*p* < 0.001). There were no significant differences in emergency admission, length of hospital stay, living alone, congestive heart failure, cerebrovascular disease, or CRP level between the groups.

**TABLE 1:** Characteristics of study participants according to whether or not the CFS score worsened from discharge to 3 months after discharge.

	Total	Worsened	Maintained/Improved	*p*
Patients	601	87 (14.5)	514 (85.5)	
Age, years	83.6 ± 6.7	83.8 ± 5.9	83.6 ± 6.8	0.827
Male sex	253 (42.1)	40 (46.0)	213 (41.4)	0.481
Emergency admission	357 (59.4)	44 (50.6)	313 (60.9)	0.077
Length of hospital stay, days	15.0 [9.0, 24.0]	13.0 [9.0, 22.0]	15.0 [9.0, 25.0]	0.110
Education, years	11.2 ± 2.8	11.9 ± 2.9	11.1 ± 2.8	0.022*
Living alone	130 (22.3)	15 (17.4)	115 (23.2)	0.264
Barthel index	90.0 [55.0, 100.0]	95.0 [79.0, 100.0]	90.0 [50.0, 100.0]	0.008*
Baseline CFS score	5.0 ± 1.7	4.4 ± 1.6	5.0 ± 1.7	0.003*
Baseline CFS score ≥ 4	467 (77.7)	61 (70.1)	406 (79.0)	0.071
CFS score at discharge	5.0 ± 1.7	4.4 ± 1.6	5.1 ± 1.7	< 0.001*
CFS score at 3 months after discharge	5.1 ± 1.7	5.7 ± 1.6	5.0 ± 1.7	< 0.001*
CCI value	2.3 ± 1.8	2.3 ± 1.8	2.3 ± 1.8	0.687
Congestive heart failure	93 (15.5)	17 (19.5)	76 (14.8)	0.263
Cerebrovascular disease	39 (6.5)	7 (8.0)	32 (6.2)	0.484
MMSE score	20.3 ± 7.7	21.1 ± 6.1	20.2 ± 8.0	0.272
GDS‐15 score	4.9 ± 3.7	5.6 ± 3.8	4.7 ± 3.6	0.047*
MNA‐SF score	9.5 ± 3.2	9.9 ± 3.1	9.4 ± 3.2	0.215
CRP, mg/dL	2.3 [0.2, 9.6]	1.4 [0.2, 6.5]	2.6 [0.2, 10.0]	0.067

*Note:* Data are shown as the number, number (percentage), mean ± standard deviation, or median [interquartile range] unless otherwise indicated. Percentages for variables with missing data were calculated using available cases.

Abbreviations: CCI, Charlson comorbidity index; CFS, clinical frailty scale; CRP, C‐reactive protein; GDS‐15, 15‐item geriatric depression scale; MMSE, mini‐mental state examination; MNA‐SF, mini nutritional assessment short form.

* Statistically significant (*p* < 0.05).

Table 2 shows the results of the logistic multiple regression analysis, with the presence or absence of worsening of the CFS score from discharge to 3 months after discharge as the objective variable. GDS‐15 score on admission was used as the predictor variable. Age and sex were added as covariates in Model 1, age, sex, and baseline CFS score in Model 2, and age, sex, baseline CFS score, MMSE score, and the CCI value in Model 3. In all models, a higher GDS‐15 score on admission was associated with significantly higher odds of CFS worsening from discharge to 3 months after discharge (Model 1: odds ratio 1.064, *p* = 0.045; Model 2: odds ratio 1.103, *p* = 0.003; Model 3: odds ratio 1.109, *p* = 0.002). Sensitivity analyses showed that the association between GDS‐15 score on admission and post‐discharge CFS worsening remained significant in models that included length of hospital stay, congestive heart failure and cerebrovascular disease, living alone, Barthel index and MNA‐SF score, or emergency admission, length of hospital stay, and log‐transformed CRP (Table S1).

**TABLE 2:** Results of multiple logistic regression analysis to identify predictors of worsening of the Clinical Frailty Scale score from discharge to 3 months after discharge.

	*B*	Odds ratio (95% CI)	*p*
Model 1			
Age (years)	0.003	1.003 (0.97–1.04)	0.886
Sex (men, 0; women, 1)	−0.212	0.809 (0.51–1.28)	0.367
GDS‐15 score	0.062	1.064 (1.00–1.13)	0.045*
Model 2			
Age (years)	0.037	1.038 (1.00–1.08)	0.074
Sex (men, 0; women, 1)	−0.096	0.909 (0.57–1.45)	0.689
Baseline CFS score	−0.332	0.717 (0.60–0.85)	< 0.001*
GDS‐15 score	0.098	1.103 (1.03–1.18)	0.003*
Model 3			
Age (years)	0.037	1.038 (1.00–1.08)	0.072
Sex (men, 0; women, 1)	−0.097	0.908 (0.56–1.46)	0.690
Baseline CFS score	−0.414	0.661 (0.53–0.82)	< 0.001*
CCI value	0.018	1.018 (0.89–1.16)	0.789
MMSE score	−0.028	0.972 (0.93–1.01)	0.188
GDS‐15 score	0.103	1.109 (1.04–1.18)	0.002*

Abbreviations: CCI, Charlson comorbidity index; CI, confidence interval; GDS‐15, 15‐item geriatric depression scale; MMSE, mini‐mental state examination.

* Statistically significant (*p* < 0.05).

A subanalysis was then performed for the frail group (baseline CFS score ≥ 4). Comparisons by background and worsening CFS score in this group are shown in Table S2. The analysis included 467 patients. The mean age was 84.9 ± 6.3 years, 183 (39.2%) were male, and the group with worsening CFS had a significantly higher education level (*p* = 0.030), higher Barthel index (*p* = 0.016), and higher GDS‐15 scores on admission (*p* = 0.017). They also had significantly lower CFS scores at baseline (*p* = 0.006) and at discharge (*p* = 0.001) and higher CFS scores at 3 months after discharge (*p* < 0.001). Table 3 shows the results of the logistic multiple regression analysis with the presence or absence of worsening of the CFS score from discharge to 3 months after discharge as the objective variable only for patients with a baseline CFS score of ≥ 4. Again, the GDS‐15 score on admission was used as a predictor variable, with age and sex added as covariates in Model 1, age, sex, and baseline CFS score in Model 2, and age, sex, baseline CFS score, MMSE score, and CCI value in Model 3. Even in analyses restricted to the frail group, a higher GDS‐15 score on admission was associated with significantly higher odds of CFS worsening in all models (Model 1: odds ratio 1.090, *p* = 0.017; Model 2: odds ratio 1.099, *p* = 0.011; Model 3: odds ratio 1.106, *p* = 0.007). A similar subanalysis was performed in the non‐frail group (baseline CFS score ≤ 3). However, multiple logistic regression analysis showed no significant association between the GDS‐15 score on admission and CFS worsening in any of the models (Table S3). A formal interaction analysis did not show a significant interaction between GDS‐15 score on admission and baseline frailty status for post‐discharge CFS worsening (odds ratio for the interaction term 0.997, *p* = 0.969; Table S4).

**TABLE 3:** Results of multiple logistic regression analysis to identify predictors of worsening of the Clinical Frailty Scale score from discharge to 3 months after discharge in patients with a baseline CFS score of ≥ 4.

	*B*	Odds ratio (95% CI)	*p*
Model 1			
Age (years)	0.011	1.011 (0.97–1.06)	0.619
Sex (men, 0; women, 1)	−0.030	0.971 (0.56–1.69)	0.917
GDS‐15 score	0.086	1.090 (1.02–1.17)	0.017*
Model 2			
Age (years)	0.029	1.029 (0.98–1.08)	0.236
Sex (men, 0; women, 1)	0.036	1.037 (0.59–1.82)	0.899
Baseline CFS score	−0.371	0.690 (0.53–0.90)	0.006*
GDS‐15 score	0.094	1.099 (1.02–1.18)	0.011*
Model 3			
Age (years)	0.028	1.029 (0.98–1.08)	0.248
Sex (men, 0; women, 1)	0.057	1.059 (0.60–1.87)	0.844
Baseline CFS score	−0.532	0.588 (0.41–0.84)	0.003*
CCI value	0.061	1.063 (0.91–1.24)	0.428
MMSE score	−0.033	0.967 (0.92–1.02)	0.187
GDS‐15 score	0.100	1.106 (1.03–1.19)	0.007*

Abbreviations: CCI, Charlson comorbidity index; CFS, clinical frailty scale; CI, confidence interval; GDS‐15, 15‐item geriatric depression scale; MMSE, mini‐mental state examination.

* Statistically significant (*p* < 0.05).

Finally, we performed a sensitivity analysis in the frail group after exclusion of patients with an MMSE score of < 15 to account for the possibility that the GDS‐15 may not be accurately assessed in patients with severe cognitive impairment. The results are shown in Table 4. In all models, consistent with the previous trends, there was a significant association between a higher GDS‐15 score on admission and worsening of the CFS score from discharge to 3 months after discharge (Model 1: odds ratio 1.098, *p* = 0.025; Model 2: odds ratio 1.114, *p* = 0.011; Model 3: odds ratio 1.130, *p* = 0.006).

**TABLE 4:** Results of multiple logistic regression analysis to identify predictors of worsening of the CFS score from discharge to 3 months after discharge for patients with a baseline CFS score of ≥ 4 after exclusion of those with an MMSE score of < 15.

	*B*	Odds ratio (95% CI)	*p*
Model 1			
Age (years)	0.020	1.020 (0.97–1.07)	0.431
Sex (men, 0; women, 1)	0.122	1.130 (0.61–2.08)	0.696
GDS‐15 score	0.093	1.098 (1.02–1.19)	0.025*
Model 2			
Age (years)	0.036	1.036 (0.98–1.09)	0.196
Sex (men, 0; women, 1)	0.147	1.158 (0.63–2.14)	0.642
Baseline CFS score	−0.332	0.718 (0.52–1.00)	0.051
GDS‐15 score	0.108	1.114 (1.03–1.21)	0.011*
Model 3			
Age (years)	0.033	1.034 (0.98–1.09)	0.249
Sex (men, 0; women, 1)	0.140	1.151 (0.61–2.18)	0.667
Baseline CFS score	−0.587	0.556 (0.38–0.82)	0.003*
CCI value	0.073	1.076 (0.91–1.28)	0.398
MMSE score	−0.121	0.886 (0.82–0.96)	0.002*
GDS‐15 score	0.122	1.130 (1.04–1.23)	0.006*

Abbreviations: CCI, Charlson comorbidity index; CFS, clinical frailty scale; CI, confidence interval; GDS‐15, 15‐item geriatric depression scale; MMSE, mini‐mental state examination.

* Statistically significant (*p* < 0.05).

## 4 Discussion

This multicenter study is the first to show that a higher GDS‐15 score on admission was associated with worsening of the CFS score from discharge to 3 months after discharge in older patients admitted to an acute care hospital. The results were consistent after adjustment for cognitive function, comorbidities, and the baseline CFS score, and also remained significant in sensitivity analyses that included length of hospital stay, congestive heart failure, and cerebrovascular disease, living alone, Barthel index, and MNA‐SF score, or emergency admission, length of hospital stay, and log‐transformed CRP. In subgroup analyses, this association was found in the frail group (baseline CFS score ≥ 4) but not in the non‐frail group (baseline CFS score ≤ 3). However, a formal interaction analysis did not show a significant interaction between GDS‐15 score and baseline frailty status; therefore, the subgroup findings should be interpreted cautiously. These findings are in line with previous findings of a close relationship between frailty and depression.

In many cases, frailty improves with inpatient care, even in older people, in comparison with the frailty status immediately before admission [5, 25]. However, how to manage the small group of patients whose frailty and physical function deteriorate after discharge has been a major challenge in clinical practice. Therefore, in this study, identification of depressive symptoms on admission as a potential marker for this adverse outcome is clinically significant. This finding highlights the importance of screening for depressive symptoms using the GDS‐15 on admission to hospital, identifying high‐risk patients early, considering interventions for depression as needed, and implementing preventive interventions for worsening frailty as needed. Patients with high GDS‐15 scores may benefit from structured post‐discharge follow‐up, including reassessment of frailty and functional status, evaluation for depressive symptoms, rehabilitation and nutritional support, medication review, and assessment of social support or isolation. Psychiatric evaluation may also be considered when clinically indicated. On the other hand, it is known that antidepressants are less effective in frail older patients and are associated with more adverse events than in younger, more robust populations [26]. Therefore, pharmacotherapy should be implemented with caution, and non‐pharmacological treatment that takes into account changes in the environments and interventions to avoid social isolation may be more important [27].

Noteworthy in this study is that the association of a higher GDS‐15 score with post‐discharge CFS worsening was observed in patients who were frail at baseline. Although the interaction analysis did not prove that baseline frailty status modified this association, this subgroup finding remains clinically meaningful because frail older patients have limited physiological reserve and are particularly vulnerable to adverse outcomes after hospitalization. One reason for this finding may be that depression and frailty share many common risk factors and yet have bidirectional effects. For example, recent research has identified chronic systemic inflammation and impaired mitochondrial function as potential causes of both frailty and depression [28]. Furthermore, chronic inflammation and elevated inflammatory cytokines adversely affect muscle mass and strength [29]. Similarly, reduced mitochondrial function is thought to contribute to reduced skeletal muscle mass and walking speed [30, 31]. Against this background, a higher GDS‐15 score on admission may identify frail hospitalized patients who are more vulnerable to post‐discharge frailty deterioration.

This study has several strengths. First, it used the change in CFS score from discharge to 3 months after discharge as the primary endpoint. It is widely known that depression worsens physical functions, such as activities of daily living [32]. However, few studies have examined changes in GDS and CFS scores over time. The CFS is a composite that measures many factors, including cognitive function, comorbidity, and self‐rated health [20], and is not just a measure of ability to perform activities of daily living. Therefore, the CFS score is more than just a measure of physical function. In our study, the group with worsened CFS had, on average, a markedly worsening CFS score, deteriorating from a mean of 4.4 ± 1.6 at discharge to 5.7 ± 1.6 at 3 months after discharge. This change corresponds, for example, to a patient who had managed to live independently from day to day deteriorating to the point that he or she requires partial assistance with indoor chores and bathing. Interestingly, the worsened group had a higher Barthel index than the maintained/improved group. This finding suggests that post‐discharge CFS worsening was not simply explained by lower baseline BADL performance. It may also reflect that patients with relatively preserved BADL had more measurable room for subsequent worsening of the CFS score. Importantly, the association between GDS‐15 score and post‐discharge CFS worsening remained significant in sensitivity analysis that included the Barthel index and MNA‐SF score. Our findings concerning deterioration of the CFS score may have a greater impact on clinical practice than in previous studies.

A second strength is that some of the study participants had poor cognitive function. In general, the older the person, the higher the risk of worsening frailty [1, 33]. It is known that frailty is more likely to worsen in patients with impaired cognitive function [34]. Moreover, it is in this high‐risk population that worsening frailty after hospitalization becomes problematic. This study is clinically relevant because it targets a population that is prone to adverse outcomes after hospitalization, which is a real problem in clinical practice. Importantly, in this population, cognitive decline itself is often associated with worse outcomes, making it difficult to address concerns that cognitive function is affecting outcomes. We have demonstrated an association between a higher GDS‐15 score on admission and post‐discharge CFS worsening by adjusting for the MMSE score. Moreover, our results were similar when patients with an MMSE score of < 15 were excluded, supporting the robustness of the association even after accounting for severe cognitive impairment. A third strength is the multicenter prospective design, which may enhance the clinical relevance and generalizability of our findings.

This study also has several limitations. First, the GDS‐15 score was assessed within 7 days of admission and may have partly reflected acute illness burden, hospitalization‐related distress, sleep disruption, pain, or other transient factors in addition to depressive symptoms. However, sensitivity analyses including emergency admission, length of hospital stay, and log‐transformed CRP showed similar results, supporting the robustness of the main finding. Second, the GDS‐15 score may not be accurate in patients with severe cognitive impairment. However, as mentioned earlier, we performed a sensitivity analysis that excluded patients with an MMSE score of < 15, which had similar results. Third, baseline CFS was assessed retrospectively at admission based on information from patients or caregivers regarding the patient's frailty status 2 weeks before admission; therefore, recall bias may have affected the baseline CFS assessment. Fourth, detailed information on admission diagnoses, disease severity, eating alone, and other social environmental factors was not available. Nevertheless, sensitivity analyses including length of hospital stay, congestive heart failure and cerebrovascular disease, living alone, and CRP showed similar results. Fifth, a very large number of patients were excluded. Therefore, the possibility of selection bias cannot be ruled out. In particular, patients with missing CFS/GDS‐15 data or inability to complete the GDS‐15 were excluded, which may have limited the generalizability of our findings to older inpatients with severe cognitive impairment or unstable clinical conditions. However, our study was conducted in the clinical setting of an inpatient acute care hospital and not in a population recruited specifically for the study. Thus, an increase in exclusions was inevitable to some extent.

## 5 Conclusions

A higher GDS‐15 score on admission was identified as an independent predictor of worsening of the CFS score from discharge to 3 months after discharge in older patients hospitalized for acute care. This association was also observed among patients with baseline CFS scores of ≥ 4 and remained significant after excluding patients with MMSE scores of < 15. Screening for depressive symptoms using the GDS‐15 on admission may be important for identifying older inpatients who require closer post‐discharge monitoring for frailty deterioration.

## Author Contributions

**Yosuke Yamada:** conceptualization, data curation, formal analysis, methodology, writing and investigation. **Hirotaka Nakashima:** data curation and validation. **Masaaki Nagae:** data curation. **Kazuhisa Watanabe:** investigation. **Chisato Fujisawa:** investigation. **Hitoshi Komiya:** investigation. **Tomihiko Tajima:** investigation. **Shosuke Satake:** investigation. **Yasushi Takeya:** investigation. **Yumi Umeda‐Kameyama:** investigation. **Hiroyuki Umegaki:** supervision, funding acquisition, and validation.

## Funding

This work was supported by a Japan Society for the Promotion of Science (JSPS) grant (JP21H02826). The funders were not involved in the study design, data collection, analysis, interpretation, or writing of the paper.

## Ethics Statement

This study was approved by the Ethics Committee of Nagoya University Hospital (approval number: 2019–0260). The study was conducted in accordance with the Declaration of Helsinki. All participants gave written informed consent.

## Conflicts of Interest

The authors declare no conflicts of interest.

## Supporting information


**Figure S1:** Study flowchart.

**Table S1:** Sensitivity analyses showing the association between GDS‐15 score and post‐discharge CFS worsening.

**Table S2:** Characteristics of patients with a baseline CFS score of ≥ 4 according to whether the CFS score worsened from discharge to 3 months after discharge.

**Table S3:** Results of multiple logistic regression analysis to identify predictors of worsening of the CFS score from discharge to 3 months after discharge in patients with a baseline CFS score of ≤ 3.

**Table S4:** Formal interaction analysis for baseline frailty status.

## Data Availability

The data that support the findings of this study are not publicly available due to their containing information that could compromise the privacy of research participants but are available from the authors (yoyamada@med.nagoya-u.ac.jp) upon reasonable request.
