# Supplementary material for: A High Geriatric Depression Scale Score on Admission to Hospital Predicts a Worse Clinical Frailty Scale Score After Discharge
Source: Geriatr Gerontol Int. 2026 Jun 30;26(7):e70598. doi: 10.1111/ggi.70598 (PMC13316975; doi:10.1111/ggi.70598)
Supplement: Supplementary file 1 — Figure S1: Study flowchart. [file GGI-26-0-s004.docx]

Supplementary Figure 1. Study flowchart

N=1848

Patients admitted and discharged between October 2019 and July 2023

Total n=1247

Met the following exclusion criteria:

(1) hospitalization of two days or less (n=50)

(2) consent not obtained (n=225)

(3) doctors estimated life expectancy to be less than one month (n=81)

(4) readmission less than 3 months since last hospitalization (n=130)

(5) transfer from other departments within the hospital

(n=175)

(6) Missing CFS or **GDS-15** data or inability to perform **GDS-15** (n=476)

(7) others (n=110)

N=601

Participants analyzed
