# Supplementary material for: A High Geriatric Depression Scale Score on Admission to Hospital Predicts a Worse Clinical Frailty Scale Score After Discharge
Source: Geriatr Gerontol Int. 2026 Jun 30;26(7):e70598. doi: 10.1111/ggi.70598 (PMC13316975; doi:10.1111/ggi.70598)
Supplement: Supplementary file 2 — Table S1: Sensitivity analyses showing the association between GDS‐15 score and post‐discharge CFS worsening. [file GGI-26-0-s003.docx]

Supplementary Table 1. Sensitivity analyses showing the association between GDS-15 score and post-discharge CFS worsening

| **Model** | **n** | **Odds ratio (95% CI) for GDS-15 score** | **P-value** |
| --- | --- | --- | --- |
| Main Model 3 | 601 | 1.109 (1.04–1.18) | 0.002* |
| Model 3 + length of hospital stay | 601 | 1.111 (1.04–1.19) | 0.002* |
| Model 3 + congestive heart failure + cerebrovascular disease | 601 | 1.108 (1.04–1.18) | 0.002* |
| Model 3 + congestive heart failure + cerebrovascular disease + length of hospital stay | 601 | 1.110 (1.04–1.19) | 0.002* |
| Model 3 + living alone | 582 | 1.108 (1.04–1.19) | 0.003* |
| Model 3 + Barthel index + MNA-SF | 575 | 1.091 (1.02–1.17) | 0.016* |
| Model 3 + emergency admission + length of hospital stay + log-transformed CRP | 520 | 1.093 (1.02–1.17) | 0.012* |

Model 3 included age, sex, baseline CFS score, CCI value, MMSE score, and GDS-15 score. Living arrangement was evaluated by adding living alone as a binary variable to Model 3. CRP was log-transformed as ln(CRP + 1); CRP values below zero were treated as missing.

*Statistically significant (P<0.05).
