# Supplementary material for: A High Geriatric Depression Scale Score on Admission to Hospital Predicts a Worse Clinical Frailty Scale Score After Discharge
Source: Geriatr Gerontol Int. 2026 Jun 30;26(7):e70598. doi: 10.1111/ggi.70598 (PMC13316975; doi:10.1111/ggi.70598)
Supplement: Supplementary file 3 — Table S2: Characteristics of patients with a baseline CFS score of ≥ 4 according to whether the CFS score worsened from discharge to 3 months after discharge. [file GGI-26-0-s002.docx]

Supplementary Table 2. Characteristics of patients with a baseline CFS score of ≥4 according to whether the CFS score worsened from discharge to 3 months after discharge

|  | **Total** | **Worsened** | **Maintained or improved** | **P-value** |
| --- | --- | --- | --- | --- |
| Patients | 467 | 61 (13.1) | 406 (86.9) |  |
| Age, years | 84.9 ± 6.3 | 85.2 ± 5.7 | 84.9 ± 6.3 | 0.719 |
| Male sex | 183 (39.2) | 24 (39.3) | 159 (39.2) | 1.000 |
| Emergency admission | 318 (68.1) | 37 (60.7) | 281 (69.2) | 0.187 |
| Length of hospital stay, days | 17.0 [11.0, 27.5] | 15.0 [11.0, 27.0] | 17.0 [11.0, 28.0] | 0.489 |
| Education, years | 10.9 ± 2.8 | 11.7 ± 3.0 | 10.8 ± 2.8 | 0.030* |
| Living alone | 98 (21.9) | 11 (18.3) | 87 (22.4) | 0.615 |
| Barthel index | 80.0 [45.0, 95.0] | 90.0 [56.0, 98.0] | 80.0 [40.0, 95.0] | 0.016* |
| Baseline CFS score | 5.6 ± 1.1 | 5.3 ± 1.0 | 5.6 ± 1.2 | 0.006* |
| CFS score at discharge | 5.7 ± 1.2 | 5.3 ± 1.0 | 5.7 ± 1.3 | 0.001* |
| CFS score at 3 months after discharge | 5.7 ± 1.3 | 6.5 ± 1.1 | 5.6 ± 1.3 | <0.001* |
| CCI value | 2.5 ± 1.8 | 2.6 ± 2.0 | 2.5 ± 1.8 | 0.778 |
| Congestive heart failure | 86 (18.4) | 16 (26.2) | 70 (17.2) | 0.110 |
| Cerebrovascular disease | 35 (7.5) | 7 (11.5) | 28 (6.9) | 0.197 |
| MMSE score | 18.9 ± 7.9 | 19.7 ± 6.2 | 18.7 ± 8.1 | 0.287 |
| GDS-15 score | 5.4 ± 3.8 | 6.5 ± 3.9 | 5.3 ± 3.7 | 0.017* |
| MNA-SF score | 8.8 ± 3.2 | 9.0 ± 3.1 | 8.7 ± 3.2 | 0.577 |
| CRP, mg/dL | 2.5 [0.2, 9.4] | 1.6 [0.2, 6.4] | 3.0 [0.2, 9.8] | 0.089 |

Data are shown as the number, number (percentage), mean ± standard deviation, or median [interquartile range] unless otherwise indicated. Percentages for variables with missing data were calculated using available cases.

CCI, Charlson Comorbidity Index; CFS, Clinical Frailty Scale; CRP, C-reactive protein; GDS-15, 15-item Geriatric Depression Scale; MMSE, Mini-Mental State Examination; MNA-SF, Mini Nutritional Assessment Short Form. *Statistically significant (P<0.05).
