# Supplementary material for: A High Geriatric Depression Scale Score on Admission to Hospital Predicts a Worse Clinical Frailty Scale Score After Discharge
Source: Geriatr Gerontol Int. 2026 Jun 30;26(7):e70598. doi: 10.1111/ggi.70598 (PMC13316975; doi:10.1111/ggi.70598)
Supplement: Supplementary file 4 — Table S3: Results of multiple logistic regression analysis to identify predictors of worsening of the CFS score from discharge to 3 months after discharge in patients with a baseline CFS score of ≤ 3. [file GGI-26-0-s005.docx]

Supplementary Table 3. Results of multiple logistic regression analysis to identify predictors of worsening of the CFS score from discharge to 3 months after discharge in patients with a baseline CFS score of ≤3

| **Model 1** | | | |
| --- | --- | --- | --- |
|  | B | Odds ratio (95% CI) | P-value |
| Age (years) | 0.048 | 1.049 (0.97-1.13) | 0.214 |
| Sex (men, 0; women, 1) | -0.474 | 0.622 (0.26-1.51) | 0.294 |
| GDS-15 score | 0.087 | 1.091 (0.93-1.28) | 0.287 |
| **Model 2** | | | |
|  | B | Odds ratio (95% CI) | P-value |
| Age (years) | 0.057 | 1.058 (0.98-1.15) | 0.162 |
| Sex (men, 0; women, 1) | -0.445 | 0.641 (0.26-1.56) | 0.327 |
| Baseline CFS score | -0.255 | 0.775 (0.39-1.53) | 0.462 |
| GDS-15 score | 0.099 | 1.104 (0.94-1.30) | 0.241 |
| **Model 3** | | | |
|  | B | Odds ratio (95% CI) | P-value |
| Age (years) | 0.061 | 1.063 (0.98-1.15) | 0.139 |
| Sex (men, 0; women, 1) | -0.597 | 0.551 (0.22-1.40) | 0.209 |
| Baseline CFS score | -0.258 | 0.773 (0.38-1.56) | 0.472 |
| CCI value | -0.141 | 0.869 (0.64-1.18) | 0.368 |
| MMSE score | -0.048 | 0.954 (0.86-1.06) | 0.371 |
| GDS-15 score | 0.084 | 1.088 (0.92-1.29) | 0.325 |

CCI, Charlson Comorbidity Index; CFS, Clinical Frailty Scale; CI, confidence interval; GDS-15, 15-item Geriatric Depression Scale; MMSE, Mini-Mental State Examination.

*Statistically significant (P<0.05).
