# Supplementary material for: A High Geriatric Depression Scale Score on Admission to Hospital Predicts a Worse Clinical Frailty Scale Score After Discharge
Source: Geriatr Gerontol Int. 2026 Jun 30;26(7):e70598. doi: 10.1111/ggi.70598 (PMC13316975; doi:10.1111/ggi.70598)
Supplement: Supplementary file 5 — Table S4: Formal interaction analysis for baseline frailty status. [file GGI-26-0-s001.docx]

Supplementary Table 4. Formal interaction analysis for baseline frailty status

| **Variable** | **B** | **Odds ratio (95% CI)** | **P-value** |
| --- | --- | --- | --- |
| GDS-15 score | 0.090 | 1.094 (0.94–1.28) | 0.262 |
| Baseline frailty status (CFS ≥4) | -0.735 | 0.479 (0.19–1.22) | 0.123 |
| GDS-15 score × baseline frailty status | -0.003 | 0.997 (0.84–1.19) | 0.969 |

The interaction model was adjusted for age, sex, CCI value, and MMSE score. Baseline frailty status was defined as a baseline CFS score of ≥4.
